# Chidamide Inhibits Acute Myeloid Leukemia Cell Proliferation by lncRNA VPS9D1-AS1 Downregulation via MEK/ERK Signaling Pathway

**DOI:** 10.3389/fphar.2020.569651

**Published:** 2020-10-19

**Authors:** Liman Lin, Yimei Que, Pingfan Lu, Huimin Li, Min Xiao, Xiaojian Zhu, Dengju Li

**Affiliations:** Department of Hematology, Affiliated Tongji Hospital, Tongji Medical College, Huazhong University of Science and Technology, Wuhan, China

**Keywords:** chidamide, VPS9D1-AS1, MEK/ERK signaling pathway, proliferation, long non-coding RNA, acute myeloid leukemia

## Abstract

Irregular histone modification and aberrant lncRNAs expression are closely related to the occurrence of tumors including acute myeloid leukemia (AML). However, the effects and specific underlying molecular mechanism of histone deacetylase inhibitors on lncRNA expression in AML cells are unclear. Here, we reported the effects of a novel histone deacetylase inhibitor Chidamide on proliferation and lncRNA expression in AML cells. Chidamide inhibited cell proliferation, blocked G1/S phase transition, and induced cell apoptosis through the caspase-dependent apoptotic pathway in AML cells. Chidamide also inhibited the formation of subcutaneous tumors. Transcriptome sequencing results showed that 1,195 lncRNAs were co-upregulated and 780 lncRNAs were co-downregulated after Chidamide treatment of SKM-1 cells and THP-1 cells. Combined with transcriptome sequencing data and the gene expression profiling interactive analysis dataset, we found that VPS9D1-AS1 expression was negatively correlated with the survival of AML patients. VPS9D1-AS1 knockdown inhibited cell proliferation, arrested cell cycle, as well as inhibited the formation of subcutaneous tumors *in vivo*. VPS9D1-AS1 overexpression had the reverse effect. Furthermore, VPS9D1-AS1 knockdown inhibited the MEK/ERK signaling pathway, and thus enhanced the inhibitory effect of Chidamide on AML cell proliferation. These findings suggested that targeted regulation of VPS9D1-AS1 might overcome the limitations of Chidamide in the treatment of AML.

## Introduction

Acute myeloid leukemia (AML) is a lethal disease. Most AML patients respond well to the “3 + 7” induction treatment regimen in combination with anthracyclines for 3 days and cytarabine for 7 days. Approximately 45%–65% of AML patients achieve complete remission; however, the recurrence rate is still high (Freeman et al., 2013). Although allogeneic hematopoietic stem cell transplantation is a curative approach, some patients cannot be transplanted due to the age factor or lack of donors. Research shows that abnormal epigenetic modifications are closely related to tumorigenesis, including in leukemia (Galm et al., 2006; Chen et al., 2010).

Histone modification is a crucial epigenetic mechanism. Histone deacetylases (HDACs) tightly control histone deacetylation. HDACs are expressed abnormally in several malignancies such as in gastric cancer, lung cancer, breast cancer, and hematological malignancies (Claus and Lubbert, 2003; Weichert et al., 2008; Minamiya et al., 2011; Linares et al., 2019). Abnormal expression of HDACs are significantly associated with advanced cancer and poor prognosis (Li and Seto, 2016). Only 2% of AML patients show HDAC gene mutations (Ceccacci and Minucci, 2016). HDACs have evolved earlier than the modification systems for histone proteins and target various non-histone substrates. Many of these non-histones are the products of oncogenes, tumor suppressor genes, or transcription factors that are important for hematopoiesis (Gregoretti et al., 2004). HDACs mediate the development of AML by interacting with aberrant oncogenic fusion proteins (PML-RARa, PLZF-RARa, and AML1-ETO) (Rego et al., 2000; Liu et al., 2006) and non-fused protein (BCL6) (Bereshchenko et al., 2002). These studies provide a theoretical foundation for AML treatment with histone deacetylase inhibitors (HDACis).

HDACis can induce cell cycle arrest, damaged cell apoptosis, autophagic cell death and myeloid leukemia cell differentiation (Ungerstedt, 2018). HDACis can inhibit various tumor cells growth and induce cells apoptosis, such as lung cancer (Zhang et al., 2019), pancreatic cancer (Zhao and He, 2015), multiple myeloma (Yuan et al., 2019). Treatment of AML with HDACis is still in clinical trials. Chidamide is a novel HDACi. Studies had shown that Chidamide could inhibit HDAC1/2/3/8/10/11(Ning et al., 2012). Currently, Chidamide has been used to treat relapsed or refractory peripheral T-cell lymphoma in China. Chidamide inhibits MDS and AML cells viability via JAK2/STAT3 signaling (Zhao et al., 2016). In addition, the cytotoxic effect of Cytarabine and Sorafenib on AML cells can be enhanced by Chidamide via regulating H3K9me3 and autophagy levels (Huang et al., 2019).

Long non-coding RNAs (lncRNAs) have more than 200 nucleotides. lncRNAs are involved in many biological functions, including imprinting, apoptosis, and cell cycle. Multiple lncRNA transcripts are independently associated with AML patients’ prognosis (Papaioannou et al., 2017; Beck et al., 2018; Papaioannou et al., 2019). Abnormal expressed of VPS9D1-AS1 occurs in various tumors which is closely correlated with disease prognosis. VPS9D1-AS1 level was relatively lower in patients with gastric cancer than in healthy control individuals. Furthermore, VPS9D1-AS1 level was related to gastric cancer patients’ disease-free survival rate (Chen et al., 2017). However, compared with normal prostate tissue or lung tissue, VPS9D1-AS1 was significantly upregulated in cancerous prostate tissue or cancerous non-small cell lung tissue (Wang et al., 2018; Han et al., 2020). These studies showed that VPS9D1-AS1 had different roles in different diseases. Recently, some studies reported that lncRNAs are closely related to epigenetic regulation and are correlated to AML patients’ prognosis. The effects and specific underlying molecular mechanism of HDACis in AML cells are unclear. Therefore, we hypothesized that HDACis interfere with the biological function of AML cells by regulating the expression of lncRNA.

To confirm our hypothesis, we analyzed the differences in lncRNA expression profile in AML cells before and after the cells were exposed to Chidamide. Here, we clarified the effect of Chidamide on AML cells. The obtained data indicated that Chidamide inhibits VPS9D1-AS1 expression and inhibits AML cells growth via the MEK/ERK signaling pathway. In addition, VPS9D1-AS1 knockdown enhanced the inhibitory effect of Chidamide on AML cell proliferation. These findings demonstrate the treatment approach of downregulating specific lncRNAs combined with administration of histone deacetylation inhibitors provides new insights into the treatment of AML.

## Materials and Methods

### Chemicals

Chidamide (HY-109015, purity: 98.01%), Z-VAD-FMK (HY-16658B, purity: 98.29%) and PD98059 (HY-12028, purity: 99.84%) were purchased from MedChemExpress (Monmouth, NJ, United States).

### Cell Culture

AML cell lines, SKM-1 and THP-1 were obtained from the China Center for Type Culture Collection (Wuhan, China). SKM-1 and THP-1 cells were cultured in RPMI 1640 containing 10% fetal bovine serum in humidified 5% CO_2_ at 37°C.

### Analysis of Cell Proliferation and Viability

AML cell lines, SKM-1 and THP-1 were obtained from the China Center for Type Culture Collection (Wuhan, China). SKM-1 and THP-1 cells were exposed to Chidamide at different concentrations for different times. Cell viability was tested with a CCK8 assay (Boster, Wuhan). Cell viability (%) = (OD treated − OD blank)/(OD control − OD blank) × 100%. The living cells were stained with carboxyfluorescein diacetate succinimidyl ester (MedChemExpress, HY-D0938). Stained cells were collected and detected with flow cytometry (BD Biosciences). The obtained data were analyzed by the ModFit LT software (v3.1, Verity Software House, Inc., Topsham, ME, United States).

### Analysis of the Cell Cycle and Apoptosis

Apoptosis was detected with an annexin V-FITC/PI apoptosis detection kit (Boster, Wuhan). Cell cycle was tested with PI staining (Beyotime Biotechnology, China). Stained cells were collected and analyzed on a FACScan flow cytometer (BD Biosciences). Data were analyzed by the FlowJo software (v10, Tree Star, Ashland, OR, United States). All the operations were performed according to the manufacturer’s instructions.

### Western Blotting Analysis

Cellular protein was isolated by lysis in RIPA buffer (Boster, Wuhan). Proteins in all samples were quantified with Bicinchoninic acid protein assay. Proteins of equal amounts from all samples were separated with SDS/PAGE gel (Bio-Rad, United States) and transferred onto PVDF membrane (Bio-Rad, United States). Bands were sealed with 5% skim milk, incubated in primary antibodies at 4°C overnight, then in secondary antibodies at room temperature for 1 h, and then examined and analyzed by using ChemiDoc™ XRS+ with Image Lab™ Software (Bio-Rad, United States). Following antibodies were purchased: anti-caspase-3 (#9662), anti-PARP (#9532), anti-Phospho-SAPK/JNK (Thr183/Tyr185) (#4688), anti-Phospho-p38 MAPK (Thr180/Tyr182) (#4511), anti-HDAC1 (#34589), anti- HDAC2 (#57156), and anti-Histone H3 (#4499) were purchased from Cell Signaling Technology (Beverly, MA, United States); anti-GAPDH (60004-1-Ig) was purchased from Proteintech Group (Chicago, IL, United States); anti-Phospho-ERK (AP0472), anti-Phospho-MEK (AP1021) and anti-CDK6 (A0705) were purchased from ABclonal Technology (Wuhan, China); anti-cyclin D1 (YT1172) was purchased from Immunoway Biotechnology Company (TX, United States); goat anti-rabbit and goat anti-mouse were obtained from Boster Biological Technology (Wuhan, China).

### Transcriptome Sequencing

Total RNA of SKM-1 and THP-1 cells was extracted with Trizol reagent before and after exposure to 1,000 nM Chidamide for 48 h. After purification, the purity of the RNA was determined, the RNA was quantified. Purified RNA was used for cDNA library preparation by KAPA Stranded mRNA-Seq Kit for Illumina® Platforms (KK8544). Finally, PCR products with a fragment size of 300–500 bp were selected for sequencing. The Illumina Hiseq X Ten sequencing platform was used for double-end sequencing with a read length of 150 bp. The effective reads were aligned to the GRch38 genome by tophat2 allowing four mismatches. The differentially expressed genes among Chidamide-treated cells and control cells were identified with the R Bioconductor package edgeR. A survival curve of co-downregulated lncRNAs was prepared from the gene expression profiling interactive analysis (GEPIA) dataset. GEPIA, a highly visual analysis website, is based on the Cancer Genome Atlas and Genotype-tissue Expression dataset projects.

### Quantitative Real-Time Polymerase Chain Reaction Analysis

The appropriately treated SKM-1 and THP-1 cells were collected and resuspended in Trizol agent for RNA extraction. cDNA was converted from 1 μg RNA using a reverse transcriptase kit. Gene expression was assessed using qRT-PCR following the instructions. Finally, the obtained data were analyzed by the 2^−ΔΔCt^ method. VPS9D1-AS1 forward, AGT​GGC​CGT​TTT​ACA​GAG​ACA, VPS9D1-AS1 reverse, CAT​GCC​AAG​CTA​CGG​GAA​GG.

### Cell Transfection

Lentiviral vectors with luciferase reporter gene were obtained from GeneChem (Shanghai, China). The specific small interfering RNA targeting VPS9D1-AS1 (5ʹ-TGG​CGT​CAG​CTC​TCT​GGA​AAT-3ʹ). VPS9D1-AS1-upregulated (VPS9D1-AS1UP) and VPS9D1-AS1-downregulated (VPS9D1-AS1KD) AML cell lines were constructed by lentiviral transfection into SKM-1 and THP-1 cell lines. The corresponding control group (negative control, NC) was transfected with an empty vector. HiteansG A or HiteansG P were used as infection enhancers for cell transfection. After transfection for 72 h, 2 μg/ml puromycin was added thrice weekly for 1 week to screen for stably transfected cells. RNA was extracted from all groups, and transfection efficiency was verified with qRT-PCR.

### Xenograft Experiments

All animal experiments were approved by the Committee on Ethics of Animal Experiments at the Huazhong University of Science and Technology. We performed all experimental procedures according to the Association for Assessment and Accreditation of Laboratory Animal Care guidelines. All mice were 4-week-old female BALB/c–nude mice purchased from Beijing Vital River Laboratory Animal Technology. SKM-1 cells (1 × 10^7^) were transplanted subcutaneously in mice. Once the tumors reached a volume of 150–200 mm^3^, the mice were randomized into two groups (*n* = 5). One group was orally administered Chidamide (25 mg/kg of body weight) dissolved in 0.2% carboxymethyl cellulose and 0.1% Tween 80 (200 μl), and the other group was orally administered 1% DMSO dissolved in 0.2% carboxymethyl cellulose and 0.1% Tween 80 (200 μl) thrice weekly for 2 weeks. Two weeks after administration, all mice were euthanized to remove the tumor. All tumors were immediately weighed, imaged and fixed with 4% paraformaldehyde and subjected to hematoxylin and eosin staining and immunohistochemistry (IHC) staining.

### Statistical Analyses

Statistical significance was analyzed by using the GraphPad Prism 7.0 software (GraphPad, La Jolla, CA, United States). Data are presented as means ± SD. The significance of differences was analyzed by using Student’s *t*-test. Pairwise multiple comparisons were analyzed by using one-way analysis of variance. *p* < 0.05 was considered statistically significant (^*^ *p* < 0.05; ^**^ *p* < 0.01; ^***^ *p* < 0.001).

## Results

### Chidamide Inhibits Acute Myeloid Leukemia Cell Proliferation *In Vitro* and *In Vivo*

**FIGURE 1:**
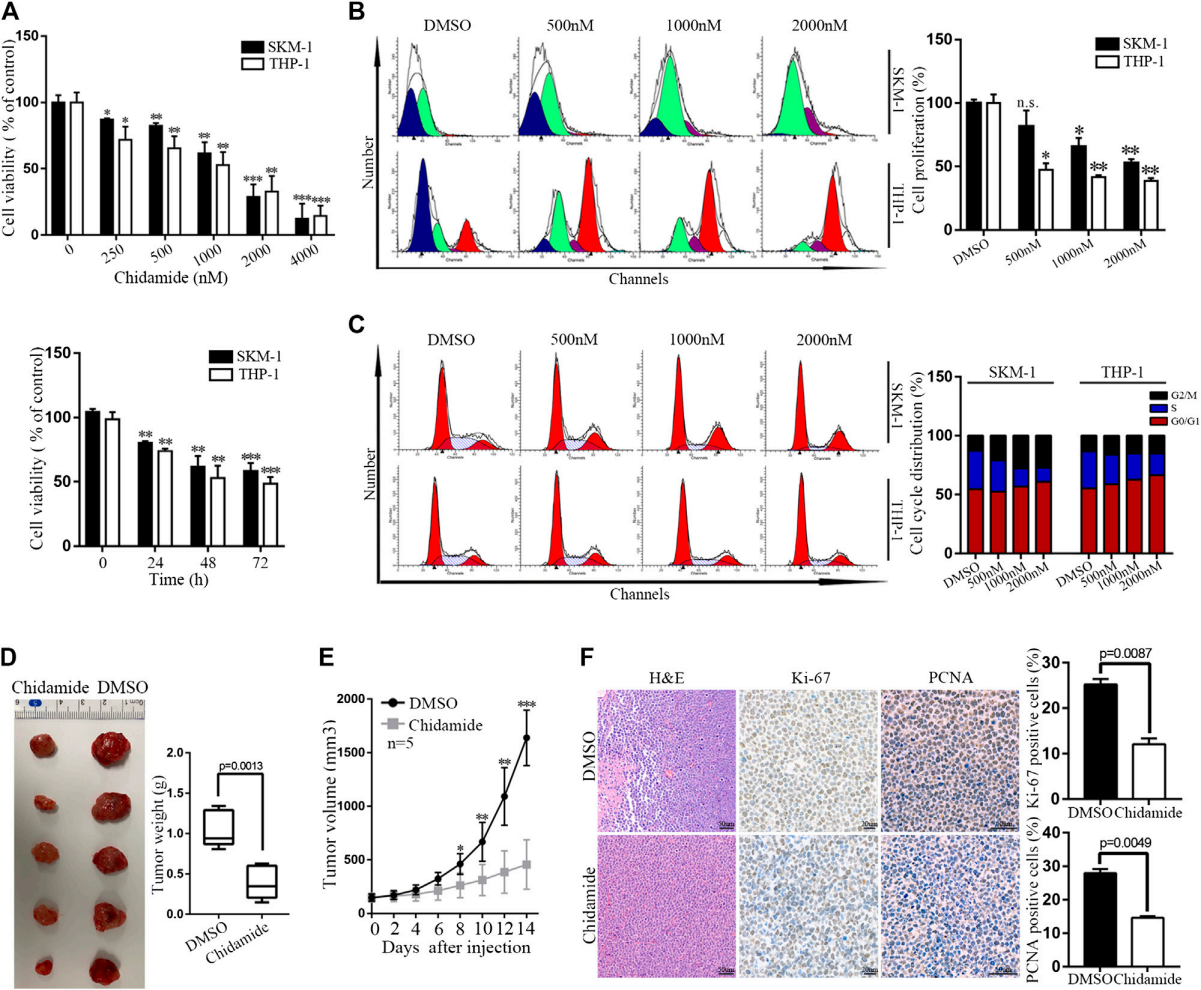
Chidamide inhibits AML cell proliferation *in vitro* and *in vivo*. **(A)** Cell viability was compared by the CCK8 assay. SKM-1 and THP-1 cells were exposed to Chidamide at different concentrations as indicated for 48 h and different times as indicated at 1,000 nM. Data are presented as mean ± SD from triplicate independent experiments ^*^ *p* < 0.05, ^**^ *p* < 0.01, ^***^ *p* < 0.001. **(B)** SKM-1 and THP-1 cells were stained with CFSE. Then cells were exposed to Chidamide at different concentrations as indicated for 48 h. ^*^ *p* < 0.05, ^**^ *p* < 0.01. **(C)** Effects of Chidamide on cell cycle progression in SKM-1 and THP-1 cells. **(D)** Images of tumors harvested from two groups of subcutaneous xenografts mice. **(E)** Tumor volume was showed when tumor volume up to 150–200 mm^3^. Tumor volume was measured once every 2 days. Data are presented as mean ± SD. ^*^ *p* < 0.05, ^**^ *p* < 0.01, ^***^ *p* < 0.001. **(F)** Images of H&E, Ki-67 (*p* = 0.0087), and PCNA (*p* = 0.0049) staining were shown in two experimental groups of tumor tissues.

Time- and dose-dependent proliferation inhibition of Chidamide were observed in AML cells (Figures 1A,B). Cell cycle analysis results showed that Chidamide treatment led to G1/S transition block (Figure 1C). We injected SKM-1 cells into upper right flanks of nude mice to construct a xenograft leukemia model. Both tumor weight and tumor size of the Chidamide-treated group were smaller than the control group (Figures 1D,E). IHC staining of xenograft tumor specimens showed that the expression levels of Ki-67 (*p* = 0.0087) and PCNA (*p* = 0.0049) in Chidamide-treated group was lower than in control group (Figure 1F).

### Chidamide Promotes Acute Myeloid Leukemia Cell Apoptosis

**FIGURE 2:**
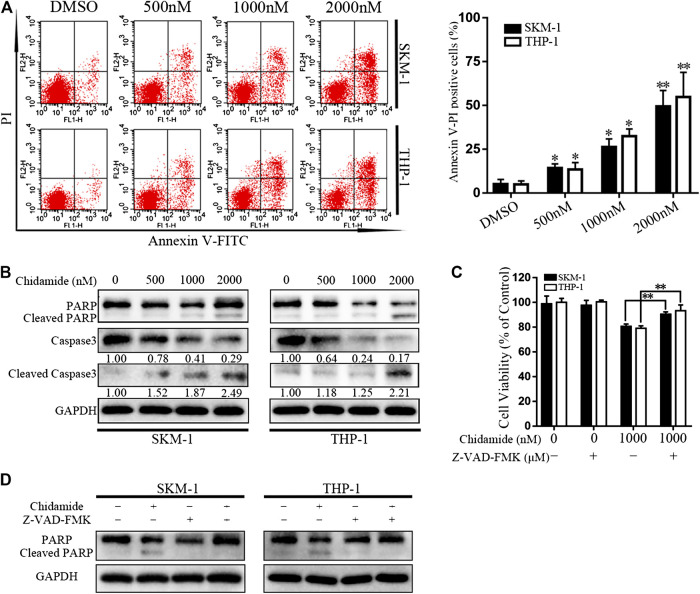
Chidamide promotes AML cell apoptosis. **(A)** Apoptotic cells were detected by flow cytometry. SKM-1 and THP-1 cells were exposed to Chidamide at indicated concentrations. ^*^ *p* < 0.05, ^**^ *p* < 0.01. **(B)** The levels of caspase-3 and PARP were detected by western blotting. Cells were treated with Chidamide for 48 h. **(C)** Cell viability was measured after cells were incubated with Chidamide (1,000 nM) and Z-VAD-FMK (50 μM) for 48 h. Data are presented as mean ± SD from triplicate independent experiments. ^*^ *p* < 0.05, ^**^ *p* < 0.01. **(D)** The levels of PARP were detected by western blotting. Cells were incubated with Chidamide (1,000 nM) and Z-VAD-FMK (50 μM) for 48 h.

After exposure to Chidamide with the specified dose for 48 h, AML cell apoptosis was induced in a dose-dependent manner (Figure 2A). Western blotting analysis showed that caspase-3 and PARP levels gradually decreased, whereas cleaved caspase-3 and cleaved PARP levels gradually increased in a concentration-dependent manner (Figure 2B). Chidamide-mediated AML cell death could be partially prevented by treatment with a pan-caspase inhibitor Z-VAD-FMK (50 μM) (*p* < 0.01) (Figure 2C). The level of cleaved PARP in response to Chidamide treatment decreased after addition of Z-VAD-FMK (Figure 2D).

### Chidamide Regulates the Expression of lncRNAs and Inhibits the Oncogenic MAPK Signaling Pathway in Acute Myeloid Leukemia Cells

**FIGURE 3:**
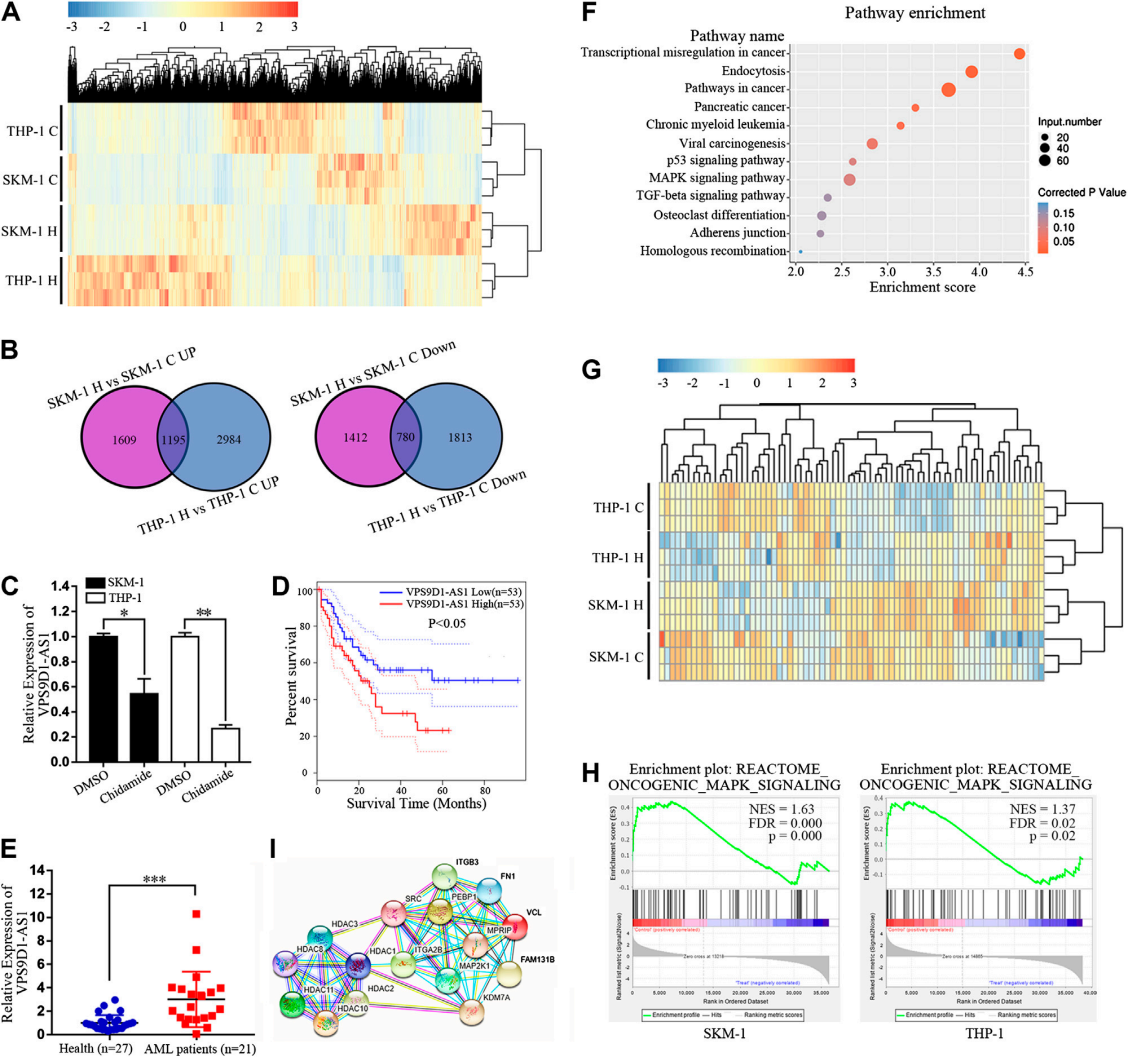
Chidamide regulates the expression of lncRNAs and inhibits the oncogenic MAPK signaling pathway in AML cells. **(A)** Cluster analysis of lncRNAs differentially expressed in Chidamide-treated group and control group. Whether the cells were treated with Chidamide at 1,000 nM for 48 h. **(B)** 1,195 lncRNAs were co-upregulated and 780 lncRNAs were co-downregulated were observed. **(C)** Expression of VPS9D1-AS1 in the Chidamide-treated group and the control group analyzed by qRT-PCR. Each bar presents mean ± SD from three independent experiments ^*^ *p* < 0.05, ^**^ *p* < 0.01. **(D)** Overall survival was analyzed and compared by Kaplan-Meier. Patients with high (lncRNA VPS9D1-AS1 high, *n* = 53) vs. low (lncRNA VPS9D1-AS1 low, *n* = 53). **(E)** Polymerase chain reaction analysis of lncRNA VPS9D1-AS1 expression in patients with *de novo* AML (*n* = 21) vs. health control people (*n* = 27). ^*^ *p* < 0.05, ^**^ *p* < 0.01, ^***^ *p* < 0.001. **(F)** Pathway enrichment analysis showing genes involved in MAPK signaling pathway were significantly enriched. **(G)** Cluster analysis was performed on genes related to the oncogenic MAPK signal. **(H)** GSEA showed that oncogenic MAPK signaling was down-regulated in Chidamide-treated AML cells. **(I)** Protein network analysis revealed top 10 of the enriched oncogenic MAPK signal proteins were closely connected to HDAC1/2/3/8/10/11.

Transcriptome sequencing was used to analyze the difference in lncRNA expression between SKM-1 and THP-1 cells before and after exposure to 1,000 nM Chidamide for 48 h. The profile of all differentially expressed lncRNAs is shown in Figure 3A. There were 4,996 differential lncRNAs in SKM-1 cells and 6,772 differential lncRNAs in THP-1 cells. The number of co upregulated lncRNAs was 1,195, whereas that of codownregulated lncRNAs was 780 (Figure 3B). Based on transcriptome sequencing data and from the GEPIA dataset, we found that 10 of the 780 codownregulated lncRNAs were associated with the survival of AML patients. Among these 10 lncRNAs, VPS9D1-AS1 was significantly downregulated after treatment with Chidamide. PCR further indicated the decreased expression of VPS9D1-AS1 in AML cells treated with 1,000 nM Chidamide for 48 h (Figure 3C). Kaplan-Meier survival analysis indicated that AML patients with higher VPS9D1-AS1 levels (*n* = 53; median survival of 10 months) had relatively shorter overall survival than those with lower levels (*n* = 53; median survival of 50 months) (Figure 3D). Additionally, VPS9D1-AS1 level was relatively higher in 22 patients with *de novo* AML than in healthy control individuals (*p* < 0.001) (Figure 3E).

We further conducted Kyoto Encyclopedia of Genes and Genomes analysis of differential lncRNAs, and results indicated significant enrichment of genes involved in the p53 signaling pathway, MAPK signaling pathway and TGF-beta signaling pathway, which are all related to cell proliferation. Among them, the number of differential genes associated with the MAPK signaling pathway was the largest (Figure 3F). Cluster analysis was performed on genes correlated with the oncogenic MAPK signal, from the RNA sequencing results (Figure 3G). Gene set enrichment analysis showed that oncogenic MAPK signaling was downregulated in Chidamide-treated AML cells (Figure 3H). Studies had shown that Chidamide could inhibit HDAC1/2/3/8/10/11(Ning et al., 2012). Protein network analysis revealed that the top 10 enriched oncogenic MAPK signal proteins were closely correlated with HDAC1/2/3/8/10/11 (Figure 3I).

### Regulation of VPS9D1-AS1 Levels Affects the Activity of AML Cells *In Vitro* and *In Vivo*

**FIGURE 4:**
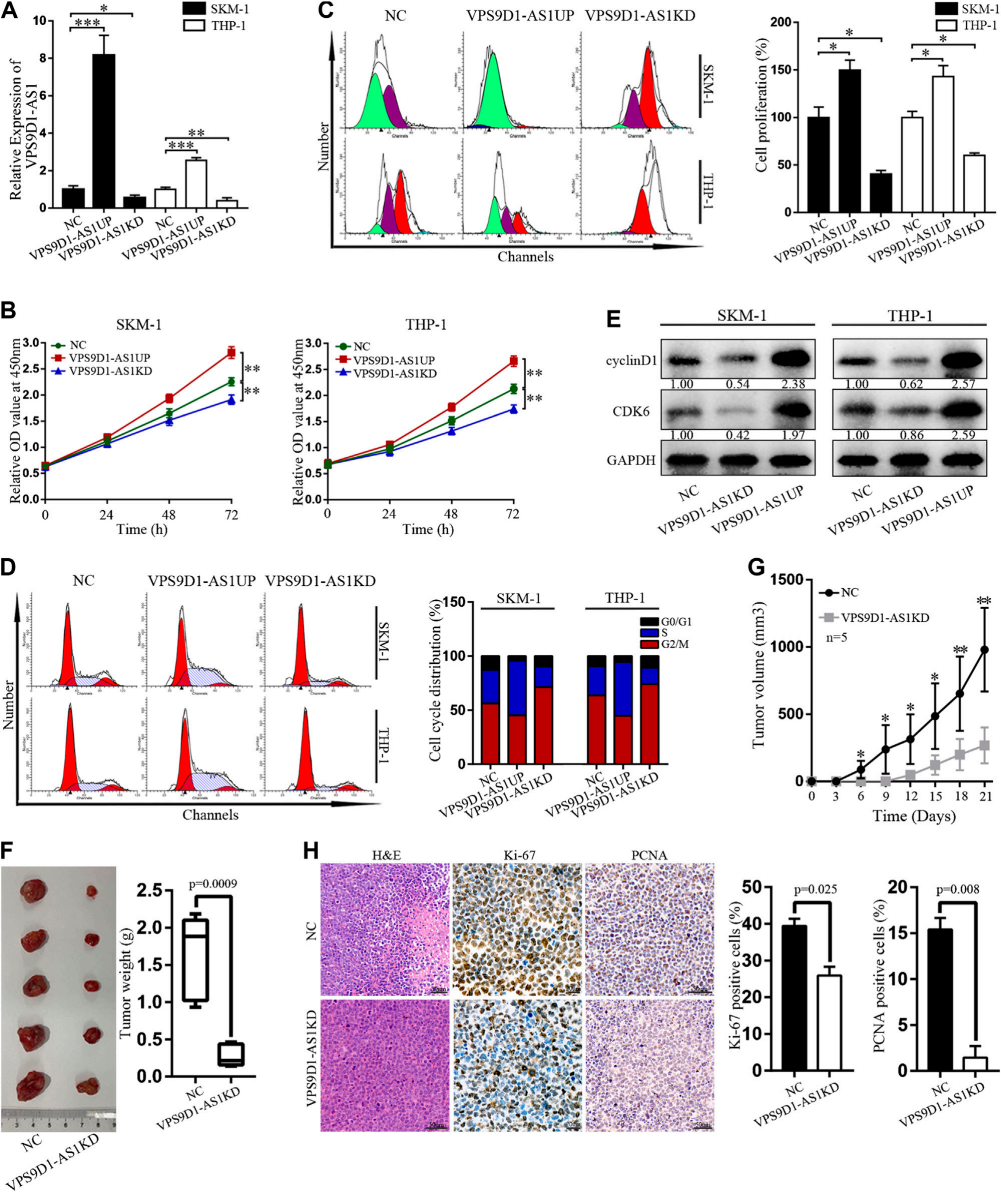
Regulation of VPS9D1-AS1 levels affects the activity of AML cells *in vitro* and *in vivo*. **(A)** Expression of VPS9D1-AS1 in stable negative control (NC), VPS9D1-AS1-upregulated (VPS9D1-AS1UP), and VPS9D1-AS1-downregulated (VPS9D1-AS1KD) analyzed by qRT-PCR. Each bar presents mean ± SD from three independent experiments ^*^ *p* < 0.05, ^**^ *p* < 0.01, ^***^ *p* < 0.001. **(B)** CCK8 assay comparing cell viability was performed in NC, VPS9D1-AS1UP, and VPS9D1-AS1KD. Data are presented as mean ± SD from triplicate independent experiments *p < 0.05, **p < 0.01. **(C)** Cell proliferation were measured by flow cytometry after staining with CFSE for 48 h. Each bar presents mean ± SD from three independent experiments ^*^ *p* < 0.05, ^**^ *p* < 0.01. **(D)** Effects of VPS9D1-AS1UP and VPS9D1-AS1KD on cell cycle progression in SKM-1 and THP-1 cells. **(E)** Western blot analyses of cell cycle protein expression in NC, VPS9D1-AS1UP, and VPS9D1-AS1KD cells. **(F)** Tumors harvesting from subcutaneous mouse xenograft were shown from two groups. **(G)** Tumor volume was measured once every 3 days. Data are presented as mean ± SD. ^*^ *p* < 0.05, ^**^ *p* < 0.01, ^***^ *p* < 0.001. **(H)** Images of hematoxylin and eosin (H&E), Ki-67 (*p* = 0.025), and PCNA (*p* = 0.008) staining were shown in tumor tissues from two groups.

Stable VPS9D1-AS1 upregulated (VPS9D1-AS1UP) and downregulated VPS9D1-AS1 (VPS9D1-AS1KD). AML cell lines were constructed by using a lentivirus transfection system. qRT-PCR was performed to determine VPS9D1-AS1 expression level and thus indicate successful construction of the cell line (Figure 4A). The proliferation of VPS9D1-AS1UP cells was higher than that of the NC cells, and the proportion of G0/G1 phase cells decreased, whereas that of S phase cells increased. Proliferation of VPS9D1-AS1KD cells was lower than that of the NC cells, and the proportion of G0/G1 phase cells increased, whereas that of the S phase cells decreased, indicating that VPS9D1-AS1 is involved in regulating AML cell growth and cell cycle (Figures 4B–D). cyclin D1 and CDK6 levels in VPS9D1-AS1KD cells were lower than in NC cells (Figure 4E). We injected VPS9D1-AS1KD and NC cells into upper right flanks of nude mice to construct a xenograft leukemia model. Both tumor weight and tumor volume of the VPS9D1-AS1KD group were smaller than the NC group (Figures 4F,G). IHC staining analysis of xenograft tumor paraffin-embedded specimens showed that the expression of Ki-67 (*p* = 0.025) and PCNA (*p* = 0.008) in the VPS9D1-AS1KD group was lower than that in the NC group (Figure 4H).

### VPS9D1-AS1 Promotes Acute Myeloid Leukemia Cell Proliferation via the MEK/ERK Signaling Pathway

**FIGURE 5:**
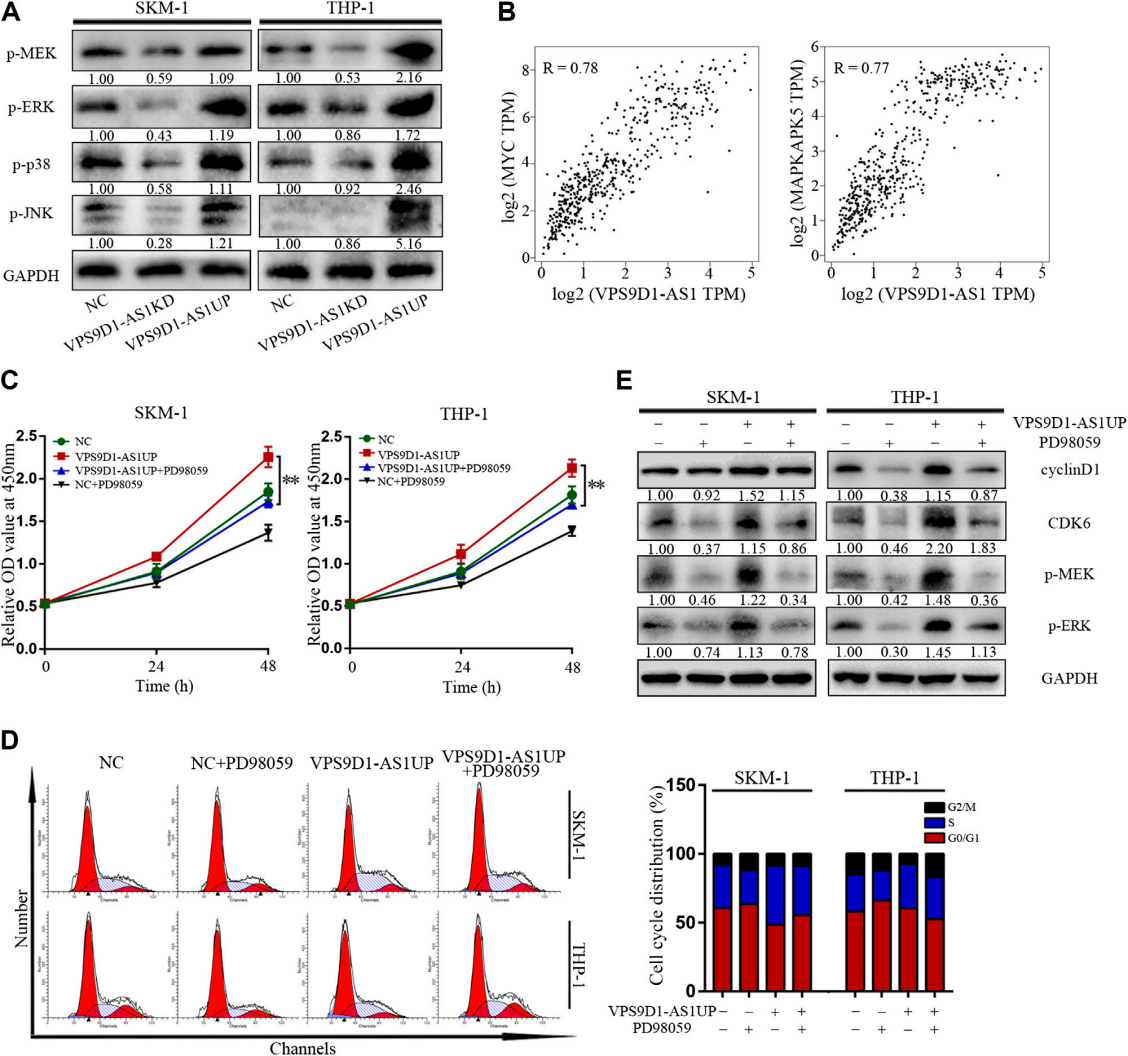
VPS9D1-AS1 promotes AML cell proliferation via the MEK/ERK signaling pathway. **(A)** Protein expression levels of key modulators of the MEK/ERK signaling pathway were analyzed by Western blotting in negative control (NC), VPS9D1-AS1-upregulated (VPS9D1-AS1UP), and VPS9D1-AS1-downregulated (VPS9D1-AS1KD) cells. **(B)** Correlation analysis between VPS9D1-AS1 and ERK/MEK signaling target genes. **(C)** Cell viability was measured after NC and VPS9D1-AS1-upregulated (VPS9D1-AS1UP) cells were treated with MEK/ERK signaling pathway inhibitor PD98059 (25 μM) for 48 h. ^*^ *p* < 0.05, ^**^ *p* < 0.01. **(D)** Cell cycle were analyzed by flow cytometry in NC and VPS9D1-AS1-upregulated (VPS9D1-AS1UP) cells were treated with PD98059 (25 μM) for 48 h. **(E)** Western blotting showed cell cycle protein expression in NC and VPS9D1-AS1-upregulated (VPS9D1-AS1UP) cells treated with PD98059 (25 μM) for 48 h.

p-MEK, p-ERK, p-p38, and p-JNK levels in VPS9D1-AS1KD cells were lower than in NC cells; however, their levels in VPS9D1-AS1UP cells were higher than in NC cells (Figure 5A). Based on the GEPIA database, multiple classic MEK/ERK signaling pathway genes are significantly related to VPS9D1-AS1. MYC and MAPKAPK5 are the top correlated genes (Figure 5B). A MEK/ERK pathway inhibitor, PD98059, inhibited VPS9D1-AS1-dependent cell proliferation and cell cycle transition and decreased cyclin D1 and CDK6 levels in VPS9D1-AS1UP cells. These results suggested that VPS9D1-AS1KD mediated inhibition of cell growth and cell cycle transition via MEK/ERK signaling pathway (Figures 5C–E).

### VPS9D1-AS1 Knockdown Enhanced the Inhibitory Effect of Chidamide

**FIGURE 6:**
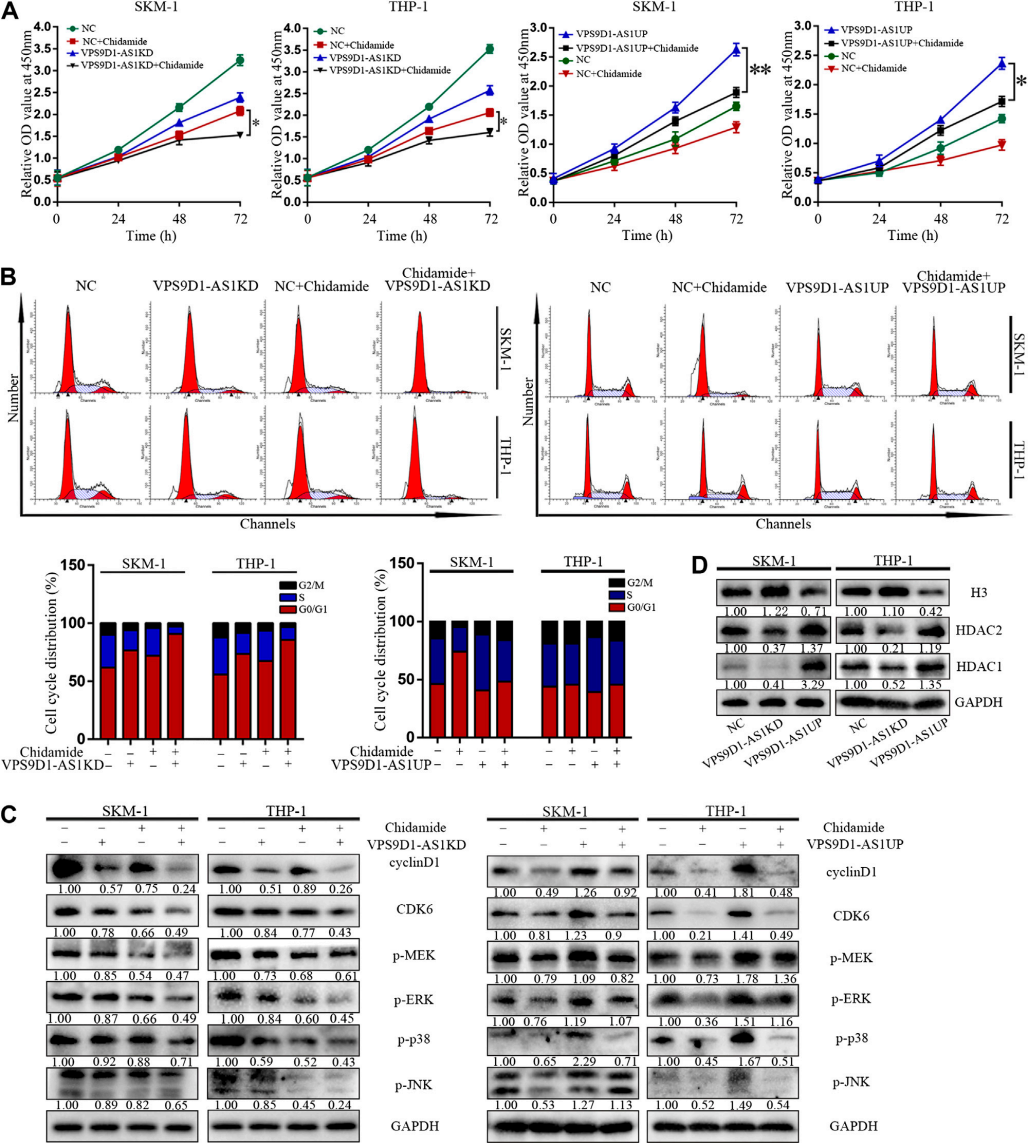
VPS9D1-AS1 knockdown enhanced the inhibitory effect of Chidamide. **(A)** CCK8 assay comparing cell viability was performed in NC, chidamide, lncRNA VPS9D1-AS1KD, lncRNA VPS9D1-AS1KD combined with chidamide, lncRNA VPS9D1-AS1UP, and lncRNA VPS9D1-AS1UP combined with chidamide (500 nM). Data are presented as mean ± SD from triplicate independent experiments (^*^ *p* < 0.05). **(B)** Effects of chidamide, lncRNA VPS9D1-AS1KD, lncRNA VPS9D1-AS1KD combined with chidamide, lncRNA VPS9D1-AS1UP, and lncRNA VPS9D1-AS1UP combined with chidamide (500 nM) on cell cycle progression in SKM-1 and THP-1 cells. **(C)** The levels of cyclin D1, CDK6, p-ERK, p-MEK, p-p38, and p-JNK in NC, chidamide, lncRNA VPS9D1-AS1KD, lncRNA VPS9D1-AS1KD combined with chidamide, lncRNA VPS9D1-AS1UP, and lncRNA VPS9D1-AS1UP combined with chidamide (500 nM) cells were detected by western blotting. **(D)** Western blot analysis of the expression of histone 3 and deacetylases in NC, VPS9D1-AS1UP and VPS9D1-AS1KD cells.

VPS9D1-AS1KD cells were significantly more sensitive to Chidamide than NC cells (*p* < 0.05) (Figure 6A). Compared with control, more number of VPS9D1-AS1KD cells treated with Chidamide remained in the G0/G1 phase, with a reduced proportion in the S phase and decreased level of cyclin D1, CDK6, p-ERK, p-MEK, p-p38 and p-JNK (Figures 6B,C). In addition, VPS9D1-AS1 increased the expression of HDAC1, HDAC2 and decreased the level of histone 3 in AML cells (Figure 6D).

## Discussion

In this study, we observed that lncRNA regulation by HDACis inhibited AML cell proliferation; this regulatory mechanism was evaluated. Results suggested that Chidamide inhibited AML cell proliferation by downregulating VPS9D1-AS1. VPS9D1-AS1 knockdown inhibited the MEK/ERK signaling pathway, and thus enhanced the inhibitory effect of Chidamide on the proliferation of AML cells.

HDACs are directly involved in modulating cellular activity and gene expression by reversing histone acetylation. Only 2% of AML patients show HDAC gene mutations (Ceccacci and Minucci, 2016). HDACs mediate the development of AML by interacting with aberrant oncogenic fusion proteins (PML-RARa, PLZF-RARa, and AML1-ETO) (Rego et al., 2000; Liu et al., 2006) and non-fused protein (BCL6) (Bereshchenko et al., 2002). HDACis can inhibit the activity of HDACs and inhibit the proliferation of various tumor cells (Marks and Xu, 2009; Jiang et al., 2019; Yuan et al., 2019; Zhang et al., 2019). Here, we explored the potential molecular mechanism of HDACis in AML cells by using Chidamide. Chidamide is a novel HDACi. Chidamide inhibits mitochondrial respiration of pancreatic cancer cells by promoting Mcl-1 degradation through the ubiquitin-proteasome pathway (He et al., 2016). Chidamide also inhibits pancreatic cancer cell proliferation and induces mitochondrial apoptosis by modulating bax/bcl-2 family proteins levels (Zhao and He, 2015). By blocking NF-kB signaling pathway, Chidamide can inhibit cell proliferation and enhance drug sensitivity in multiple myeloma (Liu et al., 2019). Chidamide inhibits MDS and AML cells viability via JAK2/STAT3 signaling (Zhao et al., 2016). In addition, the cytotoxic effect of Cytarabine and Sorafenib on AML cells can be enhanced by Chidamide via regulating H3K9me3 and autophagy levels (Huang et al., 2019).Our study showed that Chidamide inhibited cell proliferation, blocked G1/S phase transition, and induced AML cell apoptosis by activating caspase-3 and PARP.

lncRNAs participate in the biological activity of cells by interfering with the expression of DNAs, RNAs, and proteins or by combining with these molecules (Mercer et al., 2009; Kung et al., 2013). As reported, IRAIN may inhibit the activation of insulin-like growth factor type I receptor, which mediates AML cell growth and tumor progression (Sun et al., 2014). LncRNA NEAT1 inhibits AML cell growth and promotes apoptosis through modulating miR-23a-3p/SMC1A (Zhao et al., 2019). In the case of TP53 mutation, deletion, or depletion, MEG3 inhibits AML cell growth by reducing MDM2 protein level (Lyu et al., 2017). In addition, lncRNAs are closely related to epigenetic regulation. LncRNA PTPRE-AS1 recruits WD repeat domain 5 and regulates M2 macrophage activation and inflammatory diseases by epigenetic promotion of PTPRE (Han et al., 2019). Our study revealed that Chidamide inhibits the growth of AML cells by VPS9D1-AS1 downregulation. VPS9D1-AS1 was highly expressed in AML patients, and VPS9D1-AS1 promoted the proliferation of AML cells. Therefore, VPS9D1-AS1 may play an oncogenic role in AML. Our result showed that VPS9D1-AS1 knockdown inhibited cell proliferation, arrested cell cycle, as well as inhibited the formation of subcutaneous tumors *in vivo*. VPS9D1-AS1 overexpression had the reverse effect. Furthermore, VPS9D1-AS1 knockdown enhanced the inhibitory effect of Chidamide on AML cell proliferation. Chidamide could inhibit HDAC1/2/3/8/10/11(Ning et al., 2012). In this study, we found that VPS9D1-AS1 knockdown decreased the expression of HDAC1, HDAC2 and increased the level of histone 3 in AML cells. This may be one of the reasons why VPS9D1-AS1 knockdown enhanced the inhibitory effect of Chidamide on AML cell proliferation.

MAPK signaling pathway plays key role in signal transduction. The extracellular signal is transferred into intracellular via the MAPK signaling pathway. Mitogen-activated protein kinases (MAPKs) coordinate and regulate various cell activities such as gene expression, proliferation, differentiation, motility, survival, and apoptosis. ERK is an important member of the MAPK family (Pearson et al., 2001) MEK/ERK signaling pathway is the key activation mechanism of MAPKs (Kim and Choi, 2015). The MEK/ERK pathway plays an essential part in cell growth. Dysregulation of MEK/ERK pathway is characteristic of multiple human tumors. MEK/ERK signaling pathway participates in regulating cell growth, differentiation, and motility (Giordano et al., 2015). Lunghi reported that downregulation of ERK activity inhibited AML cell proliferation and induced their apoptosis (Lunghi et al., 2003). Furthermore, ERK indirectly regulates the formation of the key protein E/CDK2 complex in the active cell cycle (Keenan et al., 2001; Lents et al., 2002). In this study, we confirmed that MEK/ERK signaling pathway inhibitors can inhibit VPS9D1-AS1-induced G1/S phase transition in AML cells. Our study lacked further verification *in vivo* that VPS9D1-AS1 knockdown enhanced the inhibitory effect of Chidamide on AML cell proliferation.

Taken together, our research indicates that Chidamide inhibited cell proliferation, blocked G1/S phase transition, and induced AML cell apoptosis by activating caspase-3 and PARP. VPS9D1-AS1 was significantly downregulated after treatment with Chidamide. Overexpression of VPS9D1-AS1 was negatively related to the prognosis of AML patients. VPS9D1-AS1 knockdown inhibited cell proliferation, arrested cell cycle, as well as inhibited the formation of subcutaneous tumors *in vivo*. VPS9D1-AS1 overexpression had the reverse effect. Furthermore, VPS9D1-AS1 knockdown inhibited the MEK/ERK signaling pathway, and thus enhanced the inhibitory effect of Chidamide on the proliferation of AML cells. Thus, combining specific lncRNAs with HDACi could be a novel future strategy for AML treatment.

## Data Availability Statement

The sequencing data has been deposited into BioProject (accession: PRJNA663040).

## Ethics Statement

The animal study was reviewed and approved by Animal experiments were approved by the Institutional Animal Care and Treatment Committee of Huazhong University of Science and Technology and performed according to the Association for Assessment and Accreditation of Laboratory Animal Care guidelines.

## Author Contributions

LL and DL conceptualized this study. LL completed data curation. LL, YQ, PL, and HL conducted the investigation. YQ, PL, HL, and DL completed the methodology. DL was responsible for project administration. XZ and MX were in charge of software. LL wrote the original draft. DL Wrote, reviewed and edited the manuscript.

## Funding

This study was supported by the NSFC (81770168).

## Conflict of Interest

The authors declare that the research was conducted in the absence of any commercial or financial relationships that could be construed as a potential conflict of interest.
